# Spot Urine Protein Excretion in the First Year Following Kidney Transplantation Associates With Allograft Rejection Phenotype at 1-Year Surveillance Biopsies: An Observational National-Cohort Study

**DOI:** 10.3389/fmed.2021.781195

**Published:** 2021-11-16

**Authors:** Manca Oblak, Gregor Mlinšek, Nika Kojc, Maja Frelih, Jadranka Buturović-Ponikvar, Miha Arnol

**Affiliations:** ^1^Department of Nephrology, University Medical Centre Ljubljana, Ljubljana, Slovenia; ^2^Faculty of Medicine, University of Ljubljana, Ljubljana, Slovenia; ^3^Faculty of Medicine, Institute of Pathology, University of Ljubljana, Ljubljana, Slovenia

**Keywords:** kidney transplantation, antibody-mediated rejection, T-cell mediated rejection, donor-specific antibodies, urine protein excretion

## Abstract

**Introduction:** Urine protein excretion is routinely measured to assess kidney allograft injury, but the diagnostic value of this measurement for kidney transplant pathology remains unclear. Here we investigated whether spot urine protein excretion in the first year following transplantation associates with allograft rejection phenotype at 1-year surveillance biopsies and *de-novo* occurrence of donor-specific antibodies (DSA).

**Patients and Methods:** This prospective, observational national-cohort study included 139 non-sensitized patients who received a deceased donor kidney transplant between December 2014 and 2018. All patients received basiliximab induction and tacrolimus-based immunosuppression. Estimated protein excretion rate (ePER) was calculated monthly from spot urine protein-to-creatinine ratios. At 1-year, all recipients underwent surveillance graft biopsy and were screened for *de-novo* DSA. Screening-positive sera were subjected to single antigen bead (SAB) testing. The occurrence of *de-novo* DSA was determined based on SAB reactivity patterns using a mean fluorescence intensity threshold >1,000.

**Results:** Among the 139 study patients, 27 patients (19%) had histologic evidence of T cell-mediated rejection (TCMR), and 9 patients (7%) had histologic evidence of antibody-mediated rejection (AMR) at 1-year surveillance biopsy. One year after transplant, 19 patients (14%) developed *de-novo* DSA. Compared with patients without rejection and no *de-novo* DSA, mixed-effects linear regression analysis showed a significant difference in slope of ePER during the first year in patients with AMR and *de-novo* DSA at 1-year (46, 95% CI 25–68 mg/day/1.73 m^2^ per month and 34, 95% CI 20–49 mg/day/1.73 m^2^ per month, respectively). Patients with vascular TCMR also showed a significant difference in ePER slope over time compared with patients with non-rejection findings (31, 95% CI 9–52 mg/day/1.73 m^2^ per month). The discriminatory power of ePER for intragraft rejection processes was better in patients with AMR (AUC 0.95, 95% CI 0.90–0.99; *P* < 0.001) than in those with TCMR (AUC 0.68, 95% CI 0.59–0.79; *P* = 0.002), with 89% sensitivity and 93% specificity for proteinuria >550 mg/day/1.73m^2^.

**Conclusions:** An increase in ePER in the first year following kidney transplantation associates with AMR, vascular TCMR and *de-novo* DSA at 1-year and may be used as a non-invasive clinical marker of intragraft endothelial cell injury.

## Introduction

In recent years, antibody-mediated rejection (AMR) has been identified as the primary cause of allograft failure after kidney transplantation (1–3). This specific disease is diagnosed by means of needle biopsy. Although this invasive procedure has become safer and histologic interpretation more standardized, biopsy is usually indicated in deterioration of kidney function when allograft injury already occurs (4). Therefore, protocol biopsies have been proposed to detect changes before kidney dysfunction is apparent (5). Given that biopsy procedures are invasive, complications may occur; furthermore, sampling errors may jeopardize their diagnostic value. These shortcomings have stimulated research to identify non-invasive markers that are sufficiently diagnostic for specific transplant pathologies, and that can be used as an end point in clinical studies (6).

In patients with chronic kidney disease proteinuria is directly related to the underlying glomerular disease process and strongly associates with progression to end-stage kidney disease, with good specificity and sensitivity (7). Proteinuria is also routinely measured in kidney transplant recipients. Proteinuria, in the nephrotic range as well as lower grade, has been associated with inferior kidney transplant outcomes (8–10). Current clinical guidelines suggest that a kidney allograft biopsy should be performed when there is new onset of proteinuria or unexplained proteinuria ≥3.0 g/g creatinine or ≥3.0 g/24 h (11). However, these international guidelines are not evidence-based (evidence level 2C).

More recent data showed that proteinuria >1 g/24 h is a marker for allograft outcome with reasonable predictive accuracy, especially after the first 3 months post-transplantation (12). Although high-grade proteinuria has been related to transplant glomerulopathy and *de-novo* or recurrent glomerulonephritis (GN) (12–16), the association between low-grade proteinuria and the allograft pathology, in particular AMR, within the first year after transplantation has not been considered yet.

In this study, we aimed to assess the association and diagnostic performance of measuring proteinuria in spot urine samples during routine clinical follow-up in the first year following kidney transplantation with rejection phenotype at protocol-specified kidney biopsies and occurrence of *de-novo* donor-specific antibodies (DSA) at 1-year post-transplantation. In view of the great effect of specific diseases such as AMR on outcome after kidney transplantation, insight into the diagnostic value of proteinuria early after transplantation is particularly useful. Moreover, as many research teams are evaluating novel non-invasive biomarkers for kidney allograft injury, it is important to elucidate the diagnostic value of proteinuria measurement, a simple, inexpensive, and non-invasive marker that is already universally available.

## Patients and Methods

### Study Design

In this prospective, observational national-cohort study, we enrolled all consecutive adult recipients of a first deceased donor kidney transplant at the Department of Nephrology, University Medical Center Ljubljana between December 2014 and December 2018. All patients provided written informed consent. The National Medical Ethics Committee approved the study protocol.

### Study Participants

Between December 2014 and December 2018, 211 adult patients received a deceased donor kidney transplant at our center. Sensitized recipients with preformed DSA and patients with prior transplants (*n* = 51), dual organ transplants (*n* = 13), and patients with early allograft loss within the first 90 days after transplantation (*n* = 8) were not candidates for the study. Finally, 139 patients were included in the study. All study participants were monitored regularly during the first year according to the protocol of the transplant unit of our department: twice a week in the first month, weekly in the 2nd and 3rd month, bi-weekly in the 4th and 5th month, and monthly thereafter.

The clinical data of the cohort were prospectively collected in electronic clinical patient charts, which were used for clinical patient management as well as being linked to the database used in this study.

All patients had standard immunologic risk and received basiliximab induction and tacrolimus-based immunosuppression. Patients with immediate graft function, diabetes mellitus, or previous cardiovascular events were candidates for rapid steroid withdrawal within the first week after transplantation.

### Laboratory Assessment

Proteinuria was determined from second morning spot urine samples at month 1, and then monthly in the first year after transplantation in all study patients. Estimation of 24-h protein excretion rate (ePER, mg/day/1.73 m^2^) was obtained by multiplying protein-to-creatinine ratio (PCR) and estimated creatinine excretion rate (17, 18). Urine creatinine was measured using non-isotope-dilution mass spectrometry standardized modified Jaffe reaction (calibration traceable to IDMS). Spot urine protein was measured by pyrogallol red-molybdate complex formation using a timed endpoint method. Measurements were performed on Dimension Xpand Plus Integrated Chemistry System using manufacturer's reagents (Siemens HealthCare GmbH, Erlangen Germany). Levels of ePER in the first year were then correlated with graft rejection status, rejection phenotype, and *de-novo* DSA formation at 1 year after transplantation.

At all-time points, data on serum creatinine were collected on the same day as PCR measurements. Glomerular filtration rate was estimated (eGFR) by the Chronic Kidney Disease Epidemiology Collaboration (CKD-EPI) creatinine equation (19).

### Histologic Assessment of Biopsy Samples

In all patients, surveillance allograft biopsies were performed systematically at 1-year after transplantation. An indication kidney biopsy was considered if significant allograft dysfunction occurred before 1-year (e.g., associated with delayed graft function or an increase in serum creatinine of more than 20% from baseline without other obvious causes). Slides were stained with hematoxylin eosin, periodic acid–Schiff, and silver methenamine (Jones). An immunohistochemical C4d stain (monoclonal antibody, dilution 1:500; Quidel Corporation, Santa Clara, CA) was performed on frozen tissue. Two pathologists (NK and MF) independently reviewed all biopsies, blinded for the clinical data. The severity of histologic lesions was semiquantitatively scored according to the revised Banff 2013 criteria (20).

T cell-mediated rejection (TCMR) was reported as tubulo-interstitial (borderline and grade IA/B) or vascular (grade IIA/B). The phenotypes of AMR were classified as acute or chronic active. The diagnosis of acute AMR was based on morphologic evidence of acute tissue injury (i.e., peritubular capillaritis and/or glomerulitis) and positive C4d staining. The diagnosis of chronic AMR was based on the morphologic evidence of antibody-mediated chronic tissue injury, specifically glomerular double contours compatible with chronic glomerulopathy on light and/or electron microscopy.

At the time of protocol biopsies, systematic follow-up human leukocyte antigen (HLA) antibodies (ELISA HLA class I and class II Luminex Gen-Probe LifeCodes LSA screening) and their donor-specificity in case of positive screening (using Luminex Gen-Probe LifeCodes LSA Single Antigen Beads) were evaluated. The occurrence of *de-novo* DSA was determined based on single antigen bead reactivity patterns using a mean fluorescence intensity threshold >1,000.

### Statistical Analysis

Dichotomous variables were compared with chi-squared test and continuous variables with the Student's *t-*test or the Wilcoxon-Mann-Whitney test as appropriate. For variance analysis of continuous variables in different groups, parametric one-way ANOVA and Kruskal-Wallis test were used.

The ePER trajectories were analyzed using linear mixed model regression, with ePER values from 1 to 12 months as dependent and time and the interaction of histologic phenotype/*de-novo* DSA occurrence and time as fixed effects. Furthermore, patient-specific random effect for intercept was specified. The covariance structure was specified as an autoregressive model of the first order.

Area under the receiver operator characteristic (ROC) curve analysis was performed to evaluate the diagnostic accuracy of ePER and to calculate the specificity and sensitivity for discriminating between biopsy specimens showing AMR, TCMR, and other non-rejection findings.

All tests were two-sided and *P* < 0.05 were considered to indicate statistical significance. All analyses were performed using the SPSS statistical software (IBM SPSS statistics, version 21.0, Armonk, NY, USA).

## Results

### Study Population and Histologic Classification of Kidney Allograft Biopsy Specimens

The baseline patient, donor, and transplant-related characteristics of the study population according to histologic biopsy findings at 1-year after transplantation are provided in Table 1. Among the 139 patients, 36 (26%) had histologic evidence of allograft rejection at 1-year surveillance biopsies. Among them 27 patients (75%) were classified as having TCMR, and 9 patients (25%) were classified as having AMR. A total of 103 patients (74%) had no evidence of rejection and their biopsy findings are presented in Table 1. In ten patients (7%) early acute rejection occurred before 1-year. All patients underwent an indication biopsy due to allograft dysfunction in the first 3 months after transplantation. All rejection episodes were classified as TCMR (borderline or grade IA) and treated with pulse steroids (Table 1).

**Table 1:** Baseline characteristics of the study population according to histologic diagnosis in surveillance kidney allograft biopsies performed at 1 year after transplantation^*^.

**Variables**	**All patients (*N* = 139)**	**Rejection (*n* = 36)**	**Other findings (*n* = 103)**	***P*-value**
**Recipients**				
Age (years)	49 ± 14	50 ± 16	49 ± 13	0.86
Males (%)	101 (73)	27 (75)	74 (72)	0.72
Original kidney disease				0.45
Diabetes (%)	11 (8)	2 (6)	9 (9)	
Hypertension (%)	16 (12)	7 (19)	9 (9)	
GN (%)	41 (29)	8 (23)	33 (32)	
Polycystic (%)	16 (12)	3 (8)	13 (13)	
Pyelonephritis/reflux (%)	8 (6)	3 (8)	5 (5)	
Other/undefined (%)	27/20 (19/14)	6/7 (17/19)	21/13 (20/12)	
Time on dialysis (years)	1.9 (0.8–3.2)	2.2 (0.9–3.8)	1.8 (0.8–3.0)	0.14
Last PRA (%)	0 (0–4)	0 (0–7)	0 (0–4)	0.49
**Donors**				
Age (years)	48 ± 13	52 ± 13	46 ± 12	0.022
Expanded criteria donor (%)	39 (28)	13 (36)	26 (25)	0.21
**Transplant-related**				
HLA mismatches	2.9 ± 1.1	3.0 ± 1.1	2.8 ± 1.1	0.19
Delayed graft function (%)	28 (20)	9 (25)	19 (19)	0.40
Treatment with TAC/MMF/St	82 (59)	15 (42)	67 (65)	0.024
Treatment with TAC/MMF	57 (41)	21 (58)	36 (35)	0.014
Previous rejection^#^	10 (7)	6 (17)	4 (4)	0.029
**Biopsy diagnosis at 1-year**				
TCMR (%)		27 (75)	–	–
Borderline		8 (22)	–	–
Grade IA/B		10 (27)	–	–
Grade IIA/B		9 (25)	–	–
AMR (%)		9 (25)	–	–
Acute		7 (19)	–	–
Chronic active		2 (6)	–	–
Recurrent GN (%)^##^		–	6 (6)	–
CNI nephrotoxicity (%)		–	14 (14)	–
BKVAN (%)		–	5 (5)	–
No major abnormalities (%)		–	78 (75)	–
*De-novo* DSA at 1-year	19 (14)	11 (31)	8 (8)	0.001

*GN, glomerulonephritis; PRA, panel reactive antibodies; HLA, human leukocyte antigen; TAC, tacrolimus; MMF, mycophenolate mofetil; St, steroids; TCMR, T cell-mediated rejection; AMR, antibody-mediated rejection; CNI, calcineurin inhibitor; BKVAN, BK virus associated nephropathy*.

* *Data are presented as means ± SD, medians (interquartile ranges), or as total numbers (percentages)*.

# *The diagnosis was made based on indication biopsies performed before 1-year after transplantation. All rejection episodes were diagnosed in the first 3 months and classified as TCMR (borderline or grade IA)*.

## *All recurrent GN include recurrent IgA nephropathy*.

Patients with rejection phenotypes were transplanted from older donors and more frequently undergone rapid steroid withdrawal. In addition, 6 patients with allograft rejection at 1-year surveillance biopsy experienced early TCMR (Table 1); 4 patients had TCMR (grades IA or IIA), and 2 patients had histologic evidence of chronic active AMR. At 1-year after transplant, 19 patients (14%) developed *de-novo* DSA (4 patients' class I, and 15 patients' class II), and incidence of *de-novo* DSA occurrence was significantly higher in patents with histologic evidence of allograft rejection (Table 1).

### Kidney Allograft Histology and Proteinuria

At 1-year after transplantation, spot urine protein excretion was in the low range with a mean ePER of 318 ± 308 mg/day/1.73 m^2^. The patients were stratified into three groups according to tertiles of ePER (Table 2). eGFR did not differ significantly among patients with different levels of ePER. Greater levels of ePER were significantly associated with higher Banff histologic scores related to tubulointerstitial inflammation and microvascular injury, and patients in the highest tertile had higher incidence rates of AMR and vascular TCMR. In addition, greater levels of ePER were associated with higher c4d histologic scores, chronic glomerulopathy (cg score), and higher incidences of *de-novo* DSA occurrence at 1-year (Table 2).

**Table 2:** Graft function, Banff histologic scores, and incidences of rejection phenotypes and occurrence of *de-novo* DSA according to levels of ePER (in tertiles) at 1-year after transplantation^*^.

**Parameter at 1-year**	**Tertiles of ePER at 1-year (mg/day/1.73 m** ^**2**^ **)**			***P*-value**
	**<180** **(*n* = 46)**	**180–300** **(*n* = 45)**	**>300** **(*n* = 48)**	
ePER (mg/day/ 1.73 m^2^)	125 ± 32	222 ± 32	594 ± 308	<0.001
eGFR (ml/min/ 1.73 m^2^)	67 ± 17	69 ± 20	63 ± 21	0.139
**Banff scores (mean ± SD)**				
t score	0.15 ± 0.60	0.29 ± 0.59	0.83 ± 1.08	<0.001
i score	0.09 ± 0.35	0.20 ± 0.46	0.69 ± 0.93	<0.001
ti score	0.48 ± 0.75	0.69 ± 0.85	1.27 ± 0.92	<0.001
ptc score	0	0.02 ± 0.15	0.21 ± 0.54	0.004
g score	0.07 ± 0.32	0.04 ± 0.25	0.20 ± 0.45	0.038
v score	0.04 ± 0.29	0.02 ± 0.15	0.08 ± 0.28	0.491
ci score	0.74 ± 0.68	0.98 ± 0.72	1.17 ± 0.83	0.024
ct score	1.07 ± 0.39	1.11 ± 0.49	1.23 ± 0.56	0.239
ah score	1.35 ± 0.57	1.44 ± 0.58	1.38 ± 0.61	0.699
cg score	0.06 ± 0.32	0.02 ± 0.15	0.19 ± 0.45	0.048
c4d score	0.07 ± 0.25	0.09 ± 0.29	0.33 ± 0.83	0.030
**Rejection phenotype**				
AMR	0	1	8	<0.001
TCMR	3	10	14	0.018
Borderline	1	4	3	0.382
Grade IA/B	2	3	5	0.516
Grade IIA/B	0	3	6	0.048
*De-novo* DSA	1	4	14	<0.001

*ePER, estimated protein excretion rate, eGFR, estimated glomerular filtration rate; t, tubulitis; i, interstitial inflammation; ti, total inflammation; ptc, peritubular capillaritis; g, glomerulitis; v, intimal arteritis; ci, interstitial fibrosis; ct, tubular atrophy; ah, arteriolar hyalinosis; cg, glomerular basement membrane double contours; c4d, staining for C4d on endothelial cells of peritubular capillaries by immunofluorescence; TCMR, T cell-mediated rejection; AMR, antibody-mediated rejection; DSA, donor-specific antibodies*.

* *Data are presented as means ± SD or as total numbers*.

Among patients with no evidence of rejection, levels of ePER at 1-year were slightly higher in those with recurrent GN than in those with other non-rejection findings, but the difference was not statistically significant (268 ± 109 vs. 218 ± 22 mg/day/1.73 m^2^; *P* = 0.169).

**Figure 1:**
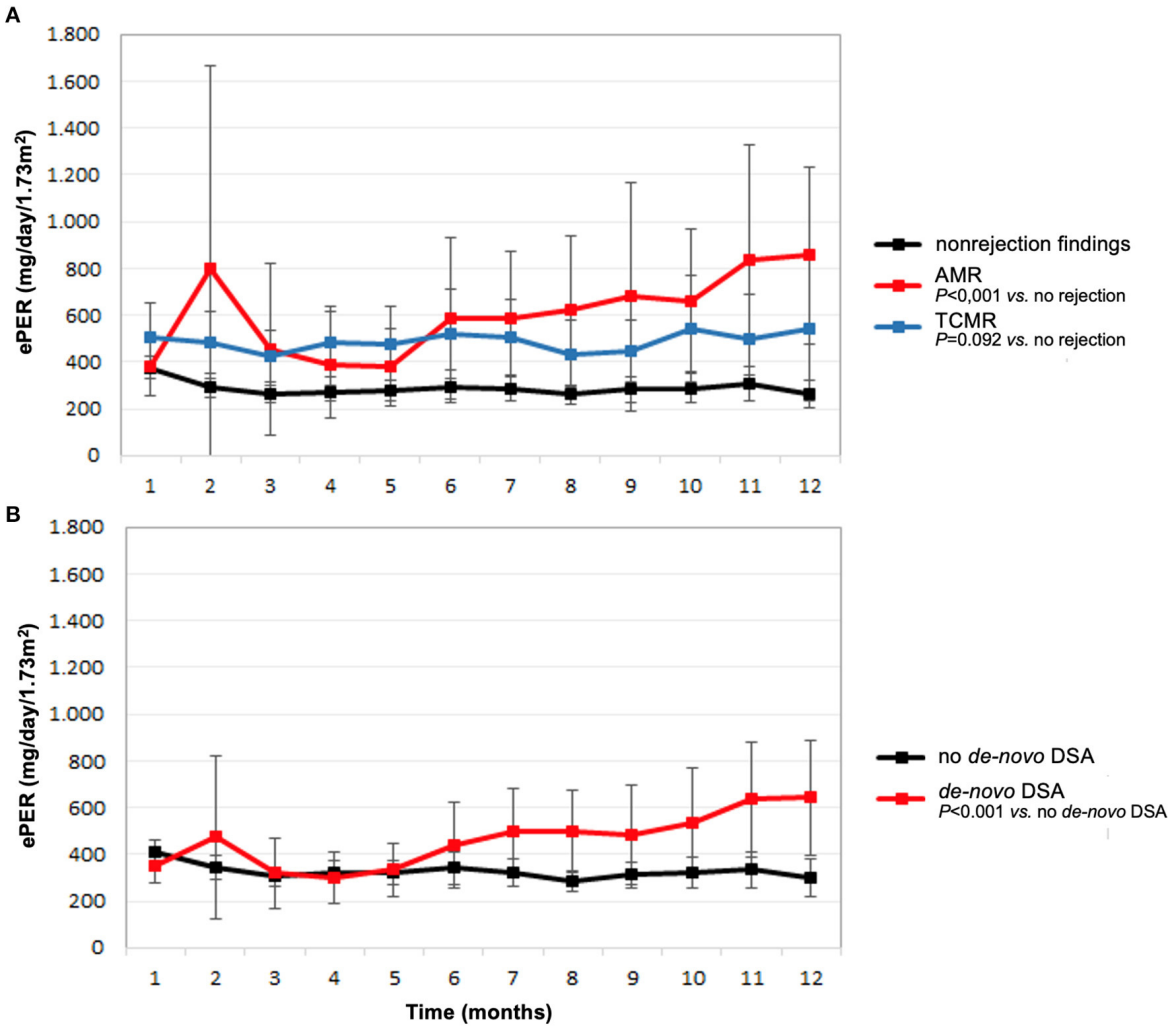
Estimated protein excretion rate (ePER, mean and 95% CI) in the first year following kidney transplantation according to main histologic findings at 1-year surveillance biopsies **(A)** and *de-novo* occurrence of donor-specific antibodies (DSA) **(B)**. AMR, antibody-mediated rejection; TCMR, T cell-mediated rejection.

The course of ePER with respect to allograft histology and occurrence of *de-novo* DSA is illustrated in Figure 1. During the 12-month period, patients with AMR, TCMR, and non-rejection findings had ePER slopes of 38 (95% confidence interval [CI] 1 to 76), 5 (95% CI −11 to 22), and −6 (95% CI −11 to −1) mg/day/1.73 m^2^ per month, respectively (Figure 1A). The difference between patients with AMR and non-rejection findings of 46 (95% CI 25–68) mg/day/1.73 m^2^ per month was statistically significant (*P* < 0.001). The difference between patients with TCMR and no rejection of 11 (95% CI −2–25) mg/day/1.73 m^2^ per month was not statistically significant (*P* = 0.092).

Patients with *de-novo* DSA had a significant increase in ePER during the first year compared with patients without *de-novo* DSA 1-year after transplantation (Figure 1B). During the 12-month period, patients with *de-novo* DSA and no *de-novo* DSA had ePER slopes of 28 (95% CI 10–46) and of −6 (95% CI −11–1) mg/day/1.73 m^2^ per month, respectively. The difference of 34 (95% CI 20–49) mg/day/1.73 m^2^ per month was statistically significant (*P* < 0.001).

**Figure 2:**
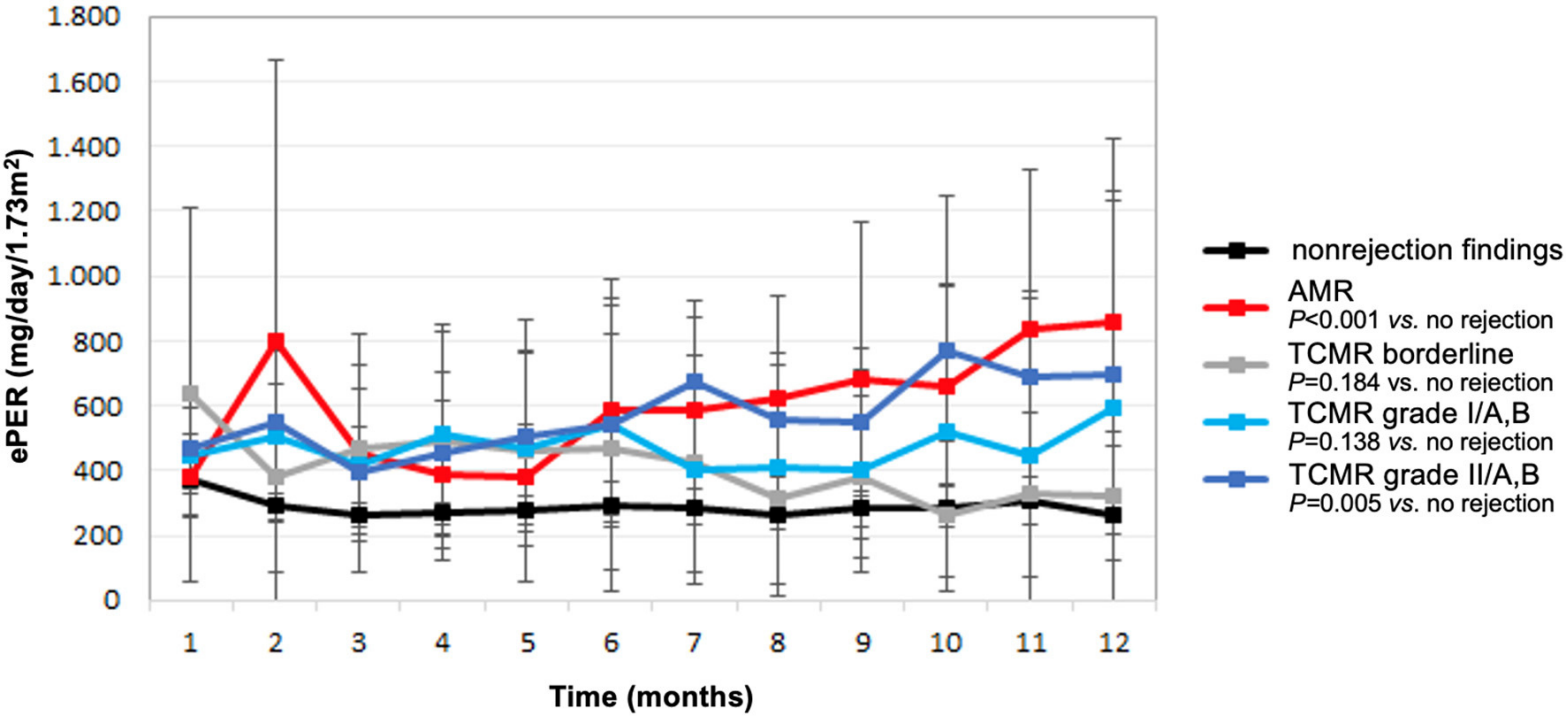
Estimated protein excretion rate (ePER, mean and 95% CI) in the first year following kidney transplantation according to allograft rejection phenotypes at 1-year surveillance biopsies. AMR, antibody-mediated rejection; TCMR, T cell-mediated rejection.

Compared with patients without evidence of rejection, ePER slopes increased progressively in patients with higher histological grade of TCMR, and the increase was highest in patients with vascular TCMR (Figure 2). During the 12-month period, patients with borderline, tubulointerstitial, and vascular TCMR had ePER slopes of −21 (95% CI, −41 to −2), 12 (95% CI, −26–51), and 24 (95% CI, −1–50) mg/day/1.73 m^2^ per month, respectively. A statistically significant difference in ePER slope was noted between patients with vascular TCMR (grades II/A,B) and no rejection (31, 95% CI 9 to 52 mg/day/1.73 m^2^ per month; *P* = 0.005). The difference between patients with borderline TCMR and no rejection (15, 95% CI −7–37 mg/day/1.73 m^2^ per month) and between patients with TCMR grades I/A,B and no rejection (15, 95% CI −5–35 mg/day/1.73 m^2^ per month) was not statistically significant (*P* = 0.184 and 0.138, respectively).

### Proteinuria as a Biomarker for Rejection-Associated Allograft Injury

Next, we examined the diagnostic performance of ePER for rejection injury phenotypes in surveillance allograft biopsies at 1-year after transplantation. ePER at 1-year post-transplant was significantly associated with the presence of AMR. The ROC AUC was 0.95 (95% CI 0.90–0.99; *P* < 0.001). The threshold of ePER that gave the maximal sensitivity and specificity for AMR was 550 mg/day/1.73 m^2^; at this threshold, the AMR can be predicted with a sensitivity of 81%, and a specificity of 80%. The diagnostic accuracy for TCMR was lower with an AUC of 0.68 (95% CI 0.59–0.79; *P* = 0.002). However, the diagnostic accuracy was better for vascular TCMR with an AUC of 0.77 (95% CI 0.66–0.89; *P* = 0.007).

## Discussion

The goal of this study was to quantify the changes in spot urine protein excretion that occur during the first year after kidney transplantation in a low-risk cohort of non-sensitized patients with stable kidney function and to investigate whether post-transplant proteinuria is associated with significant allograft pathology at 1-year. The results indicate that kidney allograft rejection and rejection phenotype at 1-year surveillance biopsies are associated with levels of ePER in the first year following transplantation. This simple diagnostic tool measured in spot urine specimens obtained longitudinally in the first-year post-transplant from patients with biopsy-confirmed tubulointerstitial TCMR and other non-rejection findings was relatively flat and distinct from the progressive increase observed in patients with AMR. In addition, *de-novo* DSA occurrence at 1-year was also associated with an increase in ePER in the first year. Moreover, ePER at 1-year was highly specific for endothelial response to injury associated with *de-novo* DSA formation, AMR and high-grade vascular TCMR. These findings are important given that spot urine protein excretion can be easily measured and followed after transplantation.

An increase in serum creatinine is often the first clinical indicator of rejection. However, it lacks sensitivity and specificity. The limitations associated with monitoring rejection by measurements of serum creatinine have been recognized previously by the observation that 30% of graft biopsies performed in patients with stable kidney function reveal histological features of rejection (21). More recently, subclinical AMR has been reported in patients with preformed anti-HLA antibodies (22). In subclinical AMR, the serum creatinine level was stable, but protocol biopsy specimens showed glomerulitis, peritubular capillary infiltration by leukocytes, and positive staining of peritubular capillaries with an anti-C4d antibody. Since AMR is associated with endothelial response-to-injury (23), one would expect an increase in urine protein excretion. Our study demonstrated that levels of proteinuria increased in the first year following transplantation among patients with histologic signs of endothelial cell injury, specifically in patients with AMR and *de-novo* occurrence of DSA. In addition, preceding TCMR in the first months after transplantation was associated with rejection phenotypes at 1-year, including chronic active AMR, as described previously (24). Therefore, persistent or increasing proteinuria may indicate ongoing rejection, even in the absence of allograft dysfunction and despite augmented immunosuppression.

Protein excretion from native kidneys falls rapidly after transplantation and *de-novo*, persistent or worsening proteinuria is usually indicative of graft pathology (25). In the largest study to date, 58% of transplanted patients with proteinuria ≥150 mg/day had transplant-specific lesions (acute rejection, transplant glomerulopathy, interstitial fibrosis/tubular atrophy) on biopsy compared with only 11% with glomerulonephritis (10). However, detailed information on the natural history of proteinuria early after transplantation and allograft injury phenotypes has not been available. In our study, we observed that in the first year following transplantation, when patients still had preserved allograft function, spot urine protein excretion was greater in patients with rejection phenotypes at 1-year surveillance biopsies. Our main observation was that proteinuria significantly increased in the patients in whom a 1-year surveillance biopsy showed AMR and in the patients who developed *de-novo* DSA. Additionally, the slope of proteinuria could discriminate between AMR and non-rejection findings. These findings fit well with recent observations of Fotheringham et al. who demonstrated that spot urine protein excretion is associated with DSA detection (26).

Next, ePER >550 mg/day/1.73 m^2^ was a specific non-invasive marker for highly relevant intragraft injury processes such as AMR and vascular TCMR in our study. The high specificity of proteinuria for these treatable diagnoses in surveillance biopsies provides the evidence of current clinical guidelines that advocate the routine measurement of proteinuria (11). In addition, clinical guidelines suggest that a kidney biopsy should be performed when there is new onset or unexplained proteinuria ≥3.0 g/g creatinine or ≥3.0 g/day. Our data illustrate that this threshold is very conservative and that early detection of proteinuria >500 mg/day could be a more sensitive threshold. However, the association between proteinuria and allograft histology was weak in the first 6 months following transplantation, likely reflecting the contribution of residual kidney function of the native kidneys in the first months (27, 28).

High specificity and sensitivity of proteinuria for AMR, vascular TCMR, and *de-novo* DSA formation demonstrate acceptable diagnostic performance of low-grade spot urine protein excretion for intragraft microcirculation inflammation and glomerular injury. Similarly, previous study from Naesens et al. (12) demonstrated that many patients with significant histologic injury had low-grade proteinuria <1 g/day, illustrating that surveillance biopsies could thus be warranted in the absence of significant proteinuria or allograft dysfunction for the timely detection of subclinical injury. In this light, our study confirmed that allograft rejection processes, specifically AMR, and *de-novo* DSA occurrence may associate with low-grade proteinuria and an increase in proteinuria already in the first year following transplantation.

The results of our study are subject to several limitations. Our strategy of including only patients with a functioning kidney beyond 90 days after transplantation may have excluded from analysis some allografts that failed early post-transplant because of rejection. This may have biased our findings toward later events. However, proteinuria in the first months after transplantation is difficult to interpret as it may originate from native kidneys or can result from injury in the grafted kidney (e.g., ischemia-reperfusion injury) (28, 29). Whether our results also apply to immunologically high-risk transplants with preformed DSA, and whether the association between the histology of AMR and proteinuria would be more pronounced in this specific high-risk patient cohort, could not be inferred from our data. Furthermore, we could not investigate the association between proteinuria in the first year and recurrent glomerulonephritis because the number of patients with recurrent disease was small and the fact that recurrence of most common glomerular diseases (e.g., IgA nephropathy) usually occurs later after transplantation. In addition, a review from Akbari et al. (30) showed that in kidney transplant population the ability of spot urine protein measurements to predict 24-h protein excretion is modest and 24-h urine collection should be considered before making further decisions. However, recent observational study showed that spot and 24-h measurements of protein excretion are similar predictors of doubling of serum creatinine, graft loss, and patient death and that spot urine samples are a suitable alternative to 24-h urine collection (31). Unfortunately, we do not have outcome data to determine whether an increase in low-grade proteinuria in the first year following transplantation associates with inferior transplant outcomes.

In conclusion, this study found that in kidney transplant recipients an increase in low-grade spot urine protein excretion in the first year following transplantation associates with AMR and *de-novo* DSA formation at 1-year post-transplant. The analysis of the diagnostic performance of low-grade proteinuria for treatable subclinical disease processes (specifically AMR, vascular TCMR, and *de-novo* DSA occurrence) in surveillance biopsies provides the scientific underpinning of the current clinical guidelines to routinely measure proteinuria early after transplantation, and to pursue a histologic diagnosis even when proteinuria >500 mg/day is detected. Further studies in an independent cohort are needed to prospectively validate these findings.

## Data Availability Statement

The datasets generated during and/or analyzed during the study are available from the corresponding author on reasonable request.

## Ethics Statement

The studies involving human participants were reviewed and approved by National Medical Ethics Committee of the Republic of Slovenia. The patients/participants provided their written informed consent to participate in this study.

## Author Contributions

MO, GM, and MA collected data. NK and MF performed histological analysis. JB-P interpreted the data and critically revised the manuscript. MA participated in the statistical analysis. All authors participated in research design, performance of the research, preparation of the manuscript, and have approved the final version of the manuscript.

## Funding

This study was funded by a research grant from the Slovenian Research Agency (ARRS ID: L3-7582) and co-funded by Astellas Pharma (Astellas ID: SI-02-RG-269). Funding sources had no role in the design and conduct of the study, collection, management, analysis, and interpretation of the data or preparation, review, or approval of this manuscript.

## Conflict of Interest

The authors declare that the research was conducted in the absence of any commercial or financial relationships that could be construed as a potential conflict of interest.

## Publisher's Note

All claims expressed in this article are solely those of the authors and do not necessarily represent those of their affiliated organizations, or those of the publisher, the editors and the reviewers. Any product that may be evaluated in this article, or claim that may be made by its manufacturer, is not guaranteed or endorsed by the publisher.

## Abbreviations

AMR: antibody-mediated rejection
CI: confidence interval
DSA: donor-specific antibodies
eGFR: estimated glomerular filtration rate
GN: glomerulonephritis
MMF: mycophenolate mofetil
PCR: protein-to-creatinine ratio
ePER: estimated protein excretion rate
ROC: receiver operator characteristic
TAC: tacrolimus
TCMR: T cell-mediated rejection.
